# Higher Peripheral Inflammation Is Associated With Lower Orbitofrontal Gamma Power in Chronic Tinnitus

**DOI:** 10.3389/fnbeh.2022.883926

**Published:** 2022-04-12

**Authors:** Linda Becker, Antonia Keck, Nicolas Rohleder, Nadia Müller-Voggel

**Affiliations:** ^1^Department of Psychology, Friedrich-Alexander Universität Erlangen-Nürnberg, Erlangen, Germany; ^2^Department of Neurosurgery, Universitätsklinikum Erlangen, Erlangen, Germany

**Keywords:** tinnitus, inflammation, MEG, C-reactive protein, stress, oscillatory activity

## Abstract

Chronic tinnitus, the continuous perception of a phantom sound, is a highly prevalent audiological symptom, for which the underlying pathology has not yet been fully understood. It is associated with neurophysiological alterations in the central nervous system and chronic stress, which can be related with a disinhibition of the inflammatory system. We here investigated the association between resting-state oscillatory activity assessed with Magnetoencephalography (MEG), and peripheral inflammation assessed by C-reactive protein (CRP) in a group of patients with chronic tinnitus (*N* = 21, nine males, mean age: 40.6 ± 14.6 years). Additionally, CRP was assessed in an age- and sex-matched healthy control group (*N* = 21, nine males, mean age: 40.9 ± 15.2 years). No MEG data was available for the control group. We found a significant negative correlation between CRP and gamma power in the orbitofrontal cortex in tinnitus patients (*p* < 0.001), pointing to a deactivation of the orbitofrontal cortex when CRP was high. No significant clusters were found for other frequency bands. Moreover, CRP levels were significantly higher in the tinnitus group than in the healthy controls (*p* = 0.045). Our results can be interpreted based on findings from previous studies having disclosed the orbitofrontal cortex as part of the tinnitus distress network. We suggest that higher CRP levels and the associated deactivation of the orbitofrontal cortex in chronic tinnitus patients is maintaining the tinnitus percept through disinhibition of the auditory cortex and attentional or emotional top-down processes. Although the direction of the association (i.e., causation) between CRP levels and orbitofrontal gamma power in chronic tinnitus is not yet known, inflammation reducing interventions are promising candidates when developing treatments for tinnitus patients. Overall, our study highlights the importance of considering immune-brain communication in tinnitus research.

## Introduction

Tinnitus is an acoustic phantom perception, defined as the subjective perception of a sound without a physical sound source. With a prevalence of up to 21% of the adult population (Fuller et al., 2020), it is a common phenomenon, which is associated with severe distress including psychiatric problems, sleep disturbances, concentration, and work impairment in 1%–3% of the population (Eggermont and Roberts, 2004; Cima et al., 2019). Clinical evidence suggests that in most cases tinnitus becomes chronic about 4 weeks after its first appearance (Wallhäusser-Franke et al., 2017). To date, no effective treatment exists mainly because processes generating and maintaining tinnitus are insufficiently understood (Langguth et al., 2019). It is widely accepted that tinnitus is initially elicited by damage of hair cells in the inner ear. Since the review by Baguley (2002) outlining that tinnitus persists after transection of the auditory nerve, it is accepted that tinnitus is generated within the central nervous system. Most research suggests that initial hearing loss triggers neuronal changes along the ascending auditory pathway leading to tinnitus. Tinnitus is associated with an increase in spontaneous activity, elevated bursting activity, reorganization of the cortical map, and an increase in neuronal synchrony (Shore et al., 2016). The role of neural synchrony is strongly supported by studies investigating abnormalities in oscillatory brain activity associated with tinnitus. At a cortical level it has been shown that oscillatory alpha activity is reduced in the auditory cortex of tinnitus patients (Weisz et al., 2005), while delta, theta, and gamma activity is increased in auditory cortical and subcortical areas (Llinas et al., 1999; Weisz et al., 2007). Counteracting pathological synchrony in the auditory system can indeed reduce tinnitus perception [e.g., increase of auditory alpha activity by neurofeedback or repetitive Transcranial Magnetic Stimulation (rTMS): Dohrmann et al., 2007; Müller et al., 2013; desynchronization of pathological brain activity and acoustic coordinated reset training: Tass et al., 2012; other TMS approaches: overview in Langguth et al., 2013]. However, results show great interindividual variability and rather small effect sizes. Furthermore, most studies did not control for hearing loss, so that it is still unclear whether changes in neuronal synchrony, especially in the high-frequency range, relate to the tinnitus percept itself or to hearing loss associated with tinnitus (Adjamian et al., 2012; Demopoulos et al., 2020).

Whether these neurophysiological alterations along the auditory pathway lead to chronic tinnitus perception, how loud or aversive tinnitus is experienced and if high psychological distress accompanies tinnitus perception depends on the co-activation of non-auditory brain networks involved in cognitive and emotional processes associated with tinnitus (de Ridder et al., 2014; Shore et al., 2016; Kleinjung and Langguth, 2020). In line with that, a multitude of studies found abnormalities in the activation of non-auditory brain regions, such as the anterior cingulate cortex (ACC), the amygdala, the insula, the (para)hippocampus and the parietal as well as the (orbitofrontal and dorsolateral) prefrontal cortex (Leaver et al., 2011; Maudoux et al., 2012; Roberts et al., 2013; McEwen et al., 2015; Rauschecker et al., 2015; Sedley et al., 2015; Minguillon et al., 2016; Mohsen et al., 2019) in tinnitus patients compared to normal-hearing controls. Accordingly, tinnitus perception and distress have been associated with aberrant oscillatory activity in low and high frequency bands in non-auditory, mainly frontal and limbic areas (Vanneste and de Ridder, 2012; Meyer et al., 2014; Lee et al., 2020).

Supporting the relevance of non-auditory influence on tinnitus, Lehner et al. (2013) revealed that multisite rTMS (prefrontal stimulation in addition to auditory stimulation) reduces tinnitus severity significantly longer (up to 3 months) compared to mere auditory stimulation.

Behavioral research emphasizes the association between perceived stress and tinnitus. During or after a period of high stress the probability to develop tinnitus increases significantly (Kleinjung and Langguth, 2020). Most interestingly, the correlation between tinnitus incidence and stress is as high as between tinnitus incidence and noise exposure (Baigi et al., 2011). Furthermore, chronic tinnitus deteriorates during exposure to stress (Langguth et al., 2007; Probst et al., 2016; Pupić-Bakrač and Pupić-Bakrač, 2020; Elarbed et al., 2021). Reducing stress through CBT, Progressive Muscle Relaxation, or Yoga can lead to reduced tinnitus severity and subjectively reported psychological distress (Weber et al., 2002; Hesser et al., 2012; Köksoy et al., 2018). Supporting the role of stress in tinnitus further, it should be emphasized that the above highlighted non-auditory regions associated with tinnitus overlap broadly with the stress network found in patients suffering from pain, functional somatic syndromes, and Posttraumatic Stress Disorder (PTSD). Such a stress network could maintain and reinforce undesired perception in tinnitus (de Ridder et al., 2011; Mohsen et al., 2019), similarly to processes observed in PTSD (Fagelson, 2007). The tied relationship between the depicted regions and stress exposure is further supported by recent studies that investigated structural neuronal changes associated with massive stress exposure. Wu et al. (2021) revealed a negative relation between the levels of perceived stress and gray matter volume of the orbitofrontal cortex, the insula and the amygdala in healthy adults indicating a detrimental effect of stress on neuronal structures.

Another line of rather recent research highlights the association between the immune system and chronic tinnitus. Tinnitus has been associated with an increased susceptibility to different physical comorbidities. A recent study by Basso et al. (2021), for instance, showed that patients with bothersome tinnitus suffer more frequently from physical comorbidities, such as cardiovascular disease, chronic shoulder pain, thyroid, or Ménière’s disease compared to non-bothersome tinnitus. A recent meta-analysis by Almufarrij and Munro (2021) states that 14.8 percent of Covid-19 patients report an onset or aggravation of tinnitus associated with their affection. The authors highlight beyond other possible causes the immune system as a potential mediator of the effect and state that, e.g., excessive production of proinflammatory cytokines may affect the audio-vestibular system (Degen et al., 2020). This is in line with evidence provided by Haider et al. (2020), who showed that the anti-inflammatory cytokine Interleukin-10 (IL-10) is significantly altered in patients suffering from chronic tinnitus compared to normal-hearing controls. Beyond, Wang et al. (2019) showed that tinnitus can be prevented by repressing the production of TNF-α (a cytokine engaged in most inflammation processes) in the auditory cortex of mice by medication. Peculiarities with regard to the immune system in tinnitus patients have also been reported in relation to stress (Mazurek et al., 2019). For example, Szczepek et al. (2014) found a positive correlation between TNF-α, perceived tinnitus loudness and stress. In line with that, Weber et al. (2002) showed that stress levels, tinnitus severity, and TNF-α levels reduced after relaxation training in tinnitus patients. These findings might be evidence for a link between inflammation processes triggered by the immune system and the above-described non-auditory, often stress-related, aspects of tinnitus.

A further immune system marker, which is related with chronic stress (Johnson and Zatorre, 2006; Juster et al., 2010) and is, therefore, highly relevant for studying the associations between chronic stress and inflammation in tinnitus, is C-reactive protein (CRP). CRP is an acute phase protein, which is synthesized in the liver. It is produced rapidly in response to inflammation, tissue damage, or vaccination and plays a key role in the innate immune system (Peisajovich et al., 2008; Perez, 2019). CRP levels are increased in patients with several diseases such as cancer, HIV, or cardiovascular diseases and elevated CRP levels are related with higher mortality (Li et al., 2017). Furthermore, there is first evidence that CRP levels are increased in chronic tinnitus (Kang et al., 2021).

Overall, it is well-known that chronic tinnitus is associated with stress and altered brain functioning. Furthermore, there is first evidence that the immune system is also altered in chronic tinnitus. Elevated immune system markers (e.g., pro-inflammatory cytokines such as IL-6 or acute phase proteins such as CRP), which are also associated with chronic stress. However, the link between brain activity and the immune system has not been investigated in chronic tinnitus so far. The aim of our study was, therefore, to investigate the association between brain activity in chronic tinnitus and the immune system (more precisely, the acute phase protein CRP).

## Methods

### Participants (Main Study, Tinnitus Sample)

Twenty-four right-handed volunteers with chronic tinnitus (duration 9.62 6 ± 9.13 years, range 6 months to 35 years) participated in the current study. They were recruited *via* flyers posted online at facebook. Three participants had to be excluded due to an excessive amount of artifacts in the MEG data (*n* = 2) or invalid CRP values (*n* = 1). The remaining* N* = 21 participants (nine males) had a mean age of 40.6 ± 14.6 years and perceived tinnitus mostly bilaterally (17 with bilateral tinnitus, three with left-sided tinnitus, and one with right-sided tinnitus). In 17 out of 20 participants (one missing), tinnitus was accompanied by hearing loss (frequency ranges: 16 kHz: *n* = 8, 8–16 kHz: *n* = 2, 4–16 kHz: *n* = 4, 2–16 kHz: *n* = 2, 2–4 kHz: *n* = 1). Tinnitus severity, assessed with the German version of Hallam’s Tinnitus Questionnaire (Goebel and Hiller, 1994), revealed a mean tinnitus severity across participants of 25.1 [range: 4 (slight) –74 (severe)]. Mean perceived stress scores, assessed with the 10-item version of the Perceived Stress Scale (PSS; Cohen et al., 1983), revealed a mean score of 24.1 ± 9.1 (two missing) for the tinnitus group.

All patients were informed about the content of the study, gave their written informed consent prior to taking part in the study and were paid 10€ per hour after participating. The Ethics Committee of the Friedrich-Alexander University of Erlangen approved the experimental procedure (protocol number: 52_17 B).

### Participants (Control Group)

Blood samples (see below) from age and sex-matched healthy controls (*N* = 21) who have participated in other studies from our lab, were used to compare CRP levels between the tinnitus patients and a healthy control group. Importantly, the blood samples were analyzed the same day as the samples from the tinnitus patients without knowing CRP levels in advance. Mean age of the control group was 40.9 ± 15.2 years, and *n* = 9 participants were male. Mean perceived stress scores, assessed with the 10-item version of the PSS revealed a mean score of 14.3 ± 5.3 for the control group.

### Experimental Procedure

The experiment was part of a bigger project on neurophysiological correlates associated with the modulation of chronic tinnitus within different experimental settings (e.g., modulation of attentional focus, relaxation, or mood). The present study focuses on the first part of the experiment, a 4-min resting state MEG-measurement with eyes open together with the assessed peripheral inflammatory marker. In the following text, we will describe only the parts relevant for the current study.

When arriving at the MEG lab, participants were informed about the study and gave their written informed consent. Participants were then fitted with head position indicators (HPI) and their individual head shapes were collected with a digitizer. After that, they were positioned supine in the MEG and instructed to keep their eyes open and to focus on a black fixation cross, presented in the middle of the screen. The experimenter then started the 4-min resting state MEG measurement. Subsequently, participants completed different experimental tasks while their brain activity was measured with MEG (e.g., relaxing vs. straining exercises with their face, listening to sounds with short gaps, attention to vs. away from tinnitus). After the actual MEG experiment, participants underwent thorough anamneses, including the assessment of perceived stress (PSS; Cohen et al., 1983), tinnitus characteristics, and severity (Goebel and Hiller, 1994).

Furthermore, to assess CRP levels, Dried Blood Spots (DBS; Danese et al., 2011; McDade, 2014) were collected at the end of the session. This method is well-suited for the assessment of CRP levels and is established in our research group (e.g., Britting et al., 2021; Becker et al., 2021a). In short, participants provided four capillary blood samples after a finger prick on a special filter paper (Whatman 903, GE Healthcare Life Sciences, Germany). The samples were dried overnight and then frozen until they were processed further. Before analysis, 3.5 mm cores were punched out and eluted overnight in phosphate buffered saline which contains 0.1% Tween 20 solution (Danese et al., 2011; McDade, 2014). The next morning, samples were shaken at 300 rpm for 1 h before further processing. The “Human C-Reactive Protein/CRP Quantikine ELISA Kit” (IBL International) was used for subsequent analysis. Absolute CRP serum concentrations were determined in duplicates using linear regression. Before statistical analysis, CRP concentrations (in μg/ml) were log-transformed to achieve a normal distribution.

### Data Acquisition With MEG

The MEG recordings were accomplished with a 248-channel whole-head-system (Magnes 3600 WH; 4D-Neuroimaging, San Diego, CA, USA) in a magnetically and electrically shielded room (Vacuumschmelze GmbH, Hanau, HE, Germany). Data were high-pass filtered online at 1 Hz and recorded with a sampling rate of 678.17 Hz. Furthermore, an online reference channel-based noise cancellation was applied. The presentation of visual stimulus material during the MEG recording was controlled using Psychopy (Peirce et al., 2019), an open-source environment for the design and control of behavioral experiments,^1^ and delivered through a mirror and projector system.

### MEG Data Analysis

MEG data analysis was performed using Matlab (The MathWorks, Natick, MA, R 2017b) and the Fieldtrip toolbox (Oostenveld et al., 2011). Aim of our analysis was to investigate whether the individual CRP level correlates with oscillatory power in specific parts of the brain. We therefore analyzed source power in six different frequency bands (delta: 1–3 Hz, theta: 4–7 Hz, alpha: 8–12 Hz, beta: 16–30 Hz, gamma low: 30–60 Hz, and gamma high: 60–90 Hz) as described in detail in the following sections.

#### Preprocessing

The raw continuous data were segmented into 2-s epochs and notch filtered at 50 Hz, 100 Hz, and 150 Hz to eliminate line noise. We then did a coarse visual artifact rejection, removing trials including any rare cases of large electromyographic (EMG) noise or technical disturbances. To minimize the influence of blinks and heartbeat related artifacts we performed an independent component analysis (ICA). Therefore, data sets were down-sampled to 150 Hz and ICA performed (RUNICA; Delorme and Makeig, 2004). The affected components were visually selected, ICA again applied to the original not down-sampled data sets and the raw data reconstructed with the respective components removed. Finally, the resulting datasets were visually inspected for remaining artifacts and residual artifactual trials rejected.

#### Source Spectral Power Analyses

Source power was assessed with a beamformer approach [Dynamic Imaging of Coherent Sources (DICS); Gross et al., 2001]. First, a template grid [using a template head model based on a segmented template MNI (Montreal Neurological Institute) brain provided by the SPM8 toolbox^2^] was created. This template grid was used to generate individual grids by warping the template grid to the individual MRIs for each participant separately. As we had no structural scans, we created “pseudo”-individual MRIs that were generated based on an initial manual co-registration of the MRI together with the individually gained surface (headshape points) and a subsequent automatic matching of the MRI head surface with the measured head surface using an iterative closest point procedure. Importantly, the obtained warped individual grids had an equal number of 6,804 points with equal positions in MNI space, so that the individual grids of different participants could be compared directly (grid points of Subject 1 correspond to grid points of Subject 2). These individual MNI grids were then utilized for creating the respective lead fields. Together, with the sensor-level cross-spectral density matrix (multitaper analysis: 2 ± 1 Hz, 5.5 ± 1.5 Hz, 10 ± 2 Hz, 23 ± 7 Hz, 45 ± 15 Hz, 65 ± 15 Hz) we could estimate spatial filters (DICS, Gross et al., 2001), optimally passing information for each grid point while attenuating influences from other regions for the particular frequency of interest. For each frequency band separately, we then applied these spatial filters to the Fourier-transformed data in the depicted frequency bands and thereby obtained source power values for each of the six frequency bands. To remove the center of the head bias we normalized power values with an estimate of the spatially inhomogeneous noise based on the smallest eigenvalue of the cross-spectral density matrix (power/noise). We then down-sampled the volumes so that, finally, we obtained power values for 21 participants, six frequency bands, and 1,917 locations distributed equally across the brain.

### Statistics

To compare CRP levels between the tinnitus sample and healthy controls, paired *T*-tests for independent samples were used. Furthermore, Pearson correlations between CRP levels and PSS scores were computed. For these analyses, the software IBM SPSS statistics (version 26 for Windows) was used.

For neurophysiological analysis, we calculated a Pearson’s correlation *T*-statistic based on the 1,917 source power values and the log-transformed CRP values for each participant and frequency band separately and tested for significant correlations across participants within the specific frequency bands using a cluster-based permutation test (Maris and Oostenveld, 2007; threshold 0.001, number of randomizations 50,000, two-sided). This analysis is testing for statistical independence between source power and behavioral data (here: individual CRP values) by randomly permuting the behavioral values. As we tested six frequency bands in parallel, we adjusted the obtained *p*-values using False Discovery Rate (Benjamini and Hochberg, 1995).

## Results

### CRP Results

Mean CRP levels were 3.6 ± 4.6 μg/ml (range 0.3–17.0 μg/ml). According to established cut-off values, CRP levels below 1 μg/ml are associated with a low, between 1 and 3 with an intermediate and >3 μg/ml with a high risk for the development of cardiovascular diseases (e.g., Blake et al., 2003; Cushman et al., 2005). In our sample, *n* = 9 were at low, *n* = 3 at intermediate, and *n* = 9 at high risk. Although not statistically significant, higher PSS scores were related with higher CRP levels in the tinnitus group (*r*_(19)_ = 0.40, *p* = 0.092).

### Comparison With the Control Group

**Figure 1:**
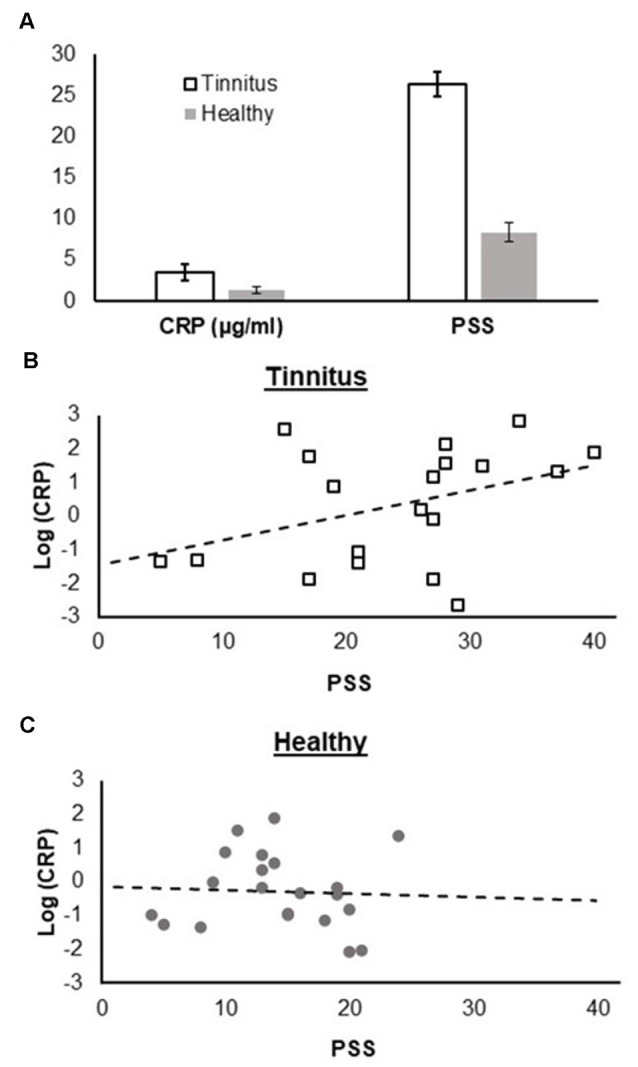
**(A)** Comparison of C-reactive protein (CRP) levels and Perceived Stress Scale (PSS) scores between tinnitus patients and healthy controls, **(B)** association between CRP levels and PSS scores in the tinnitus group, and **(C)** in the control group. CRP levels and PSS scores are scaled in the same way for both groups and panels **(B)** and **(C)** are directly comparable.

Additionally, we compared CRP levels with an age and sex-matched healthy control sample. In the control group, *n* = 14 were at low, *n* = 4 at intermediate, and *n* = 3 high risk for development of cardiovascular diseases. Mean CRP levels were significantly higher in the tinnitus group than in the control group (*t*_(25.4)_ = 2.07, *p* = 0.045; Figure 1). Moreover, PSS scores were significantly lower in the healthy controls than in the tinnitus group (*t*_(38)_ = 4.17, *p* < 0.001). Interestingly, and contrary to the tinnitus group, we found no association between PSS scores and CRP levels in the control group (*r*_(21)_ = −0.05, *p* = 0.830). An additional analysis, in which we pooled the tinnitus and the control group, revealed a small significant correlation between CRP levels and PSS scores (*r*_(40)_ = 0.33, *p* = 0.037).

### Neurophysiological Results

**Figure 2:**
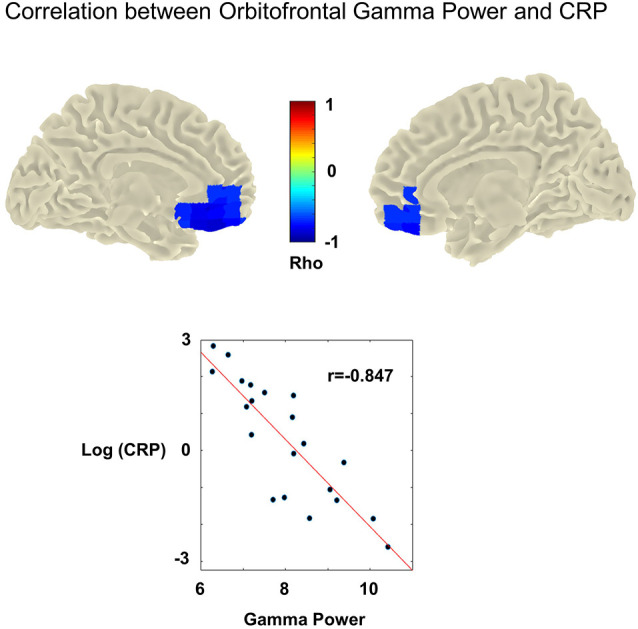
Upper panel: cluster statistic showing a significant correlation between high gamma power (60–90 Hz) and C-reactive protein levels (CRP) across participants with chronic tinnitus (cluster-*p* = 0.001, Benjamini-Hochberg adjusted *p*-value = 0.006). No MEG data was available for the control group. In the tinnitus group, high CRP levels are associated with significantly reduced gamma power (60–90 Hz). This effect is strongest in the left and right orbitofrontal cortices (most prominent correlation in left and right A11 lateral). Lower panel: association between CRP [y-axis: log(CRP)] and mean gamma power (x-axis: Gamma power/noise estimate) retrieved from the obtained significant cluster (averaged over the left and right hemisphere). Dots indicate the individual participants. The red line denominates the least-squares line. Higher CRP values are correlated significantly with reduced gamma power (Pearson’s correlation rho = −0.847,cluster-p = 0.001, Benjamini-Hochberg adjusted p-value = 0.006).

We could reveal a significant negative correlation between high gamma power (60–90 Hz) and CRP level (*r* = −0.847, cluster-*p* = 0.001, Benjamini-Hochberg adjusted *p*-value = 0.006). This correlation was most pronounced over left and right orbitofrontal cortices (left and right A11 lateral, Brainnetome Atlas; Fan et al., 2016; Figure 2). Note that for this analysis, the pooled gamma power over the left and right orbitofrontal cortex was used. Additional analyses for the left and right orbitofrontal cortex revealed the same results, i.e., negative correlations between high gamma power and CRP levels (left: −0.822, right: −0.829). For, delta, theta, alpha, beta, and low gamma power, we could not reveal significant correlations.

## Discussion

The aim of our study was to investigate the association between resting state MEG brain activity and CRP levels in chronic tinnitus. We found a significant negative correlation between high gamma and CRP levels in the BA11 lateral cluster for the tinnitus group. This indicates that low orbitofrontal high gamma power was associated with high CRP. No significant clusters were found for other frequency bands (i.e., delta, theta, alpha, beta, and low gamma).

The decrease of high gamma power in the orbitofrontal cortex when CRP is high can be interpreted as a deactivation of the orbitofrontal cortex associated with high CRP levels. This interpretation is supported by studies showing that the level of high gamma power correlates positively with the BOLD response (Logothetis et al., 2001) and is closely related to the activation of neuronal populations in the orbitofrontal cortex (Rich and Wallis, 2017).

The orbitofrontal cortex is involved in higher order cognitive functions such as sensory inhibition (Ben Shalom and Bonneh, 2019), top-down attentional control (Kam et al., 2021), and emotional regulation (Rolls, 2017). In line with that, orbitofrontal dysfunction has been associated with problems in emotion regulation or impulsive control (fear processing: Hsieh and Chang, 2020; PTSD: Franz et al., 2020; depression: Davidson et al., 2002; methamphetamine dependence: Paulus et al., 2002; Attention-Deficit-Hyperactivity Disorder: Toplak et al., 2005). Moreover, a deactivation of the prefrontal cortex has been related to reduced positive affect (Kringelbach, 2005) and an increase in pain perception (Moont et al., 2011). A deactivation of the orbitofrontal cortex in tinnitus is, therefore, very likely to reflect top-down processes maintaining tinnitus through disinhibition of auditory perception (Ben Shalom and Bonneh, 2019), attentional processes (Kam et al., 2021), or emotional reinforcement (Rolls, 2017). Most interestingly, the orbitofrontal cortex is part of the tinnitus distress network (de Ridder et al., 2011; Elgoyhen et al., 2015; Leaver et al., 2016). Further support for the relationship between a dysfunction of the orbitofrontal cortex and tinnitus comes from voxel-based morphometry, e.g., Mühlau et al. (2006) who showed that the orbitofrontal gray-matter volume is reduced in patients suffering from chronic tinnitus compared to healthy controls. In line with that, Müller et al. (2013) found that the decrease of orbitofrontal gamma power is associated with an increase in tinnitus loudness. We hypothesize that the orbitofrontal cortex could be a major hub in transferring inflammation processes to the tinnitus network and thereby stimulate central neuronal processes that maintain tinnitus. This notion is supported by a study showing that taking Naltrexone, which reduces CRP levels and acts on μ-opioid receptors in the orbitofrontal cortex (amongst other regions) led to a significant reduction of tinnitus distress (Vanneste et al., 2013).

Interestingly, perceived stress was significantly higher in the tinnitus group compared to the control group and an association between CRP and PSS score was only found in the tinnitus group (as well as in the pooled sample). This could mean that, in tinnitus patients, chronic stress triggers inflammatory processes maintaining tinnitus in the central nervous system. Such a mechanism could explain how the above-described tight association between tinnitus and stress is operating in the brain and must be part of future research.

However, due to our cross-sectional design, the direction of the association (i.e., causation) between CRP levels and orbitofrontal gamma power remains unclear. Both directions are conceivable, i.e., inflammation could either cause or exacerbate the tinnitus symptoms, or intrusive tinnitus could raise CRP levels. For instance, hypofunction of the orbitofrontal cortex could disinhibit the tinnitus symptoms, leading to greater distress, which might impact upon stress and sleep, and thereby increasing inflammation. Moreover, a bi-directional or recursive relationship would also be possible. Nevertheless, our study highlights the importance of considering immune-brain communication in tinnitus research.

Our study is subject to some further limitations, of which the most important one is that we did not record MEG data from the control group. However, the significant differences in CRP levels and PSS scores between both groups which were both related with gamma power suggest that the pattern will be different for the controls. This must be investigated in future studies. Moreover, we would like to mention here that, due to the explorative nature of our study, we decided to use rigid statistics capable of testing predominantly large effects across the whole brain. This could have kept us blind for smaller effects in other brain regions or frequency bands. A more fine-grained examination of effects in other frequency bands, brain areas or communication patterns should be addressed in future studies.

Our study provides a number of starting points for future research. The first important next step is replicating our findings within a controlled design. Because our control group was not matched with regards to hearing loss, this should also be addressed in future studies. Moreover, the underlying mechanisms as well as the direction of the association between inflammation and gamma power in the orbitofrontal cortex and the association with stress (including psychological distress as well as physiological stress) must be investigated by means of longitudinal studies.

Despite the still open questions regarding directionality, our findings have important implications for the treatment of tinnitus because they suggest that inflammation-reducing treatments might be suitable. One possibility would be physical activity interventions, which have been shown to be well-suited for reducing CRP levels (Kaltenegger et al., 2021). In general, physical activity has been shown to be suitable for reducing distress, improve mental well-being, and change re-activity of biological stress systems (e.g., Fox, 1999; Huang et al., 2013; Becker et al., 2021b). A potential alternative would be drug treatments which reduce CRP levels, but very few drugs can reduce CRP without treating the underlying pathology (Pepys and Hirschfield, 2003). Overall, there is currently no gold standard for the treatment of chronic tinnitus and not every treatment can reduce the symptoms in every tinnitus patient. One reason for this is that the underlying pathology has not yet been fully understood in any case (Langguth et al., 2019). Our results, which emphasize the role of inflammation, make an important contribution in this direction and it should be investigated in future research whether the suggested inflammation-reducing treatments are indeed also suitable to decrease the tinnitus symptoms.

## Conclusions

Our findings indicate that people with chronic tinnitus have higher CRP levels than healthy controls, which makes them particularly vulnerable. Moreover, our study highlights the role of the orbitofrontal cortex in chronic tinnitus and the importance of considering immune-brain communication in tinnitus research. Most importantly, our study emphasizes the potential of considering inflammation as part of the treatment of chronic tinnitus.

## Data Availability Statement

The original contributions presented in the study are included in the article, further inquiries can be directed to the corresponding author.

## Ethics Statement

The studies involving human participants were reviewed and approved by Ethics Committee of the Friedrich-Alexander University of Erlangen. The patients/participants provided their written informed consent to participate in this study.

## Author Contributions

LB, NR, and NM-V contributed to conception and design of the study. AK collected the data. LB and NM-V analyzed the data and wrote the first draft of the manuscript. All authors contributed to the article and approved the submitted version.

## Conflict of Interest

The authors declare that the research was conducted in the absence of any commercial or financial relationships that could be construed as a potential conflict of interest.

## Publisher’s Note

All claims expressed in this article are solely those of the authors and do not necessarily represent those of their affiliated organizations, or those of the publisher, the editors and the reviewers. Any product that may be evaluated in this article, or claim that may be made by its manufacturer, is not guaranteed or endorsed by the publisher.

## Footnotes

^1^ www.psychopy.org

^2^ http://www.neuromatic.thinkrandom.com
